# Exploring episodic specificity induction on divergent thinking in children

**DOI:** 10.1371/journal.pone.0341294

**Published:** 2026-03-06

**Authors:** Guillermo Tomás, Teresa Bajo, Alejandra Marful

**Affiliations:** Department of Experimental Psychology, Mind, Brain and Behaviour Research Center (CIMCYC), University of Granada, Granada, Spain; Public Library of Science, UNITED KINGDOM OF GREAT BRITAIN AND NORTHERN IRELAND

## Abstract

Previous studies suggest that Episodic Specificity Induction (ESI) improves the recall of episodic details and facilitates transfer to other cognitive tasks requiring episodic thinking (i.e., divergent thinking). However, the only study examining an adapted future-oriented ESI in children has failed to show benefits in subsequent cognitive tasks. To investigate this, two experiments were conducted using the standard ESI protocol with children. Experiment 1 tested second graders, fifth graders, and young adults using children-adapted materials (i.e., TV cartoons), while Experiment 2 tested fifth graders using non-adapted materials. Both experiments confirmed that ESI improved the recall of episodic details compared to a control condition. Additionally, developmental differences in episodic recall in Experiment 1 disappeared after controlling for total verbal production, suggesting that children’s episodic memory benefits when recalling materials that are child-friendly. Conversely, unexpected findings regarding transfer effect to divergent thinking revealed no transfer effects in Experiment 2 (non-adapted materials) and a significant increase in idea fluency and flexibility following the control condition in Experiment 1 (children-adapted materials). This result may be explained by a positive mood induction, as general questions accompanied by child-friendly videos could enhance creative performance following the control condition. These findings highlight the importance of carefully selecting and adapting ESI materials to children population in future studies.

## Introduction

Episodic memory can be defined as people’s ability to recall personal specific events from the past and mentally travel to the time of their occurrence [1]. It plays a central role in many daily activities that require remembering what, where, or when something was experienced [2]. From a developmental perspective, several studies have examined how episodic memory develops from childhood to adulthood [3,4]. While basic forms of episodic memories have been observed in infants (ten-week-old) [5] and toddlers [6–9], these early memories typically lack autonoetic consciousness (i.e., the ability to mentally represent our specific past experiences while being aware that it is our self-experienced memory [10]) [11,12]. In this regard, research suggests that children develop autonoetic consciousness between the ages five and seven, enabling children to mentally travel back in time [13–17]. Neuroscientific research also suggests that, during middle childhood, the development of episodic memory follows a trajectory that parallels the maturation of the prefrontal cortex and hippocampus [18–24].

Interestingly, some studies have found developmental differences in the episodic details remembered by children across middle childhood and early adolescence [25–28]. For example, Coughlin et al. conducted a study [26] with five-, seven-, nine-year-old children and, young adults in which they had to respond to an Episodic Thinking Interview, a task in which participants have to generate personal events given a word cue (i.e., cake). The narratives provided in this interview were scored according to their episodicity (from very general non-episodic details to episodic specific details situated in time and space). Their results suggested that narratives from five- and seven-year-old children were significantly less episodic than those provided by nine-year-old children and adults. Similar findings have been reported in other developmental studies in which the episodicity of the events increases within each time-period across middle childhood. For example, Gott & Lah [27] found that eight-year-old children provided fewer episodic details on the Adapted Child Autobiographical Interview than did fourteen-year-old adolescents. Likewise, Piolino et al. [28] found that participants from seven and eight years old showed poorer performance compared to eleven to thirteen-year-old children when they had to recall episodic memories of a personal event from three different periods in their past.

These developmental changes in episodic memory during childhood are important because of the direct relationship between episodic memory and other cognitive processes (encompassed by the term ‘episodic thinking’) such as prospection (i.e., simulation of future events), counterfactual thinking (i.e., simulation of events that might have happened in the past), fictional thinking (i.e., daydreaming about events that will never happen), or divergent thinking (i.e., the ability to generate a greater number of ideas to solve a problem) [3]. In this context, the constructive episodic simulation hypothesis suggests that people need to access and recombine their memories in order to imagine future scenarios [29] or to generate new and original ideas [30].

On this basis, Madore et al. [31] developed a procedure called Episodic Specificity Induction (ESI) to determine the extent to which participants improved different aspects of their episodic thinking (i.e., imagining the future, performing a creative task, etc.) by performing a brief episodic memory training before the critical task. The ESI procedure is based on the cognitive interview (protocol used in forensic and legal psychology to optimize the memory of witnesses) [32] and consists of an interview conducted after watching a short video in which participants are asked to episodically recall many details of the context, people and actions in the video. The use of this procedure has been shown to increase the amount of episodic detail recalled by participants compared to a control condition in which participants are asked general questions about the video [33]. Along these lines, many studies have shown that the enhancement of episodic memory by ESI carries over to other tasks, such as recalling autobiographical events [31,34], imagining future events [31,35], or generating new ideas in divergent thinking tasks [36,37].

Most studies on the effects of ESI have focused on young and older adults. However, there is limited research on how ESI impacts other cognitive tasks in childhood. While no studies have employed the standard ESI procedure on children, a relevant study conducted by Tanguay et al. [38] trained six- and seven-year-old children using an adapted version of ESI to future thinking (children had to imagine as many details as possible if they “would have breakfast tomorrow”) and then tested its possible effects on wide range of cognitive tasks (i.e., Prospective memory task, Recollection/imagination task, Delay of gratification task and Picture-book task). Similar to other adult experiments, children showed an increase in the number of internal (“on task”) details collected during the adapted ESI interview. However, there was not transfer effect to other cognitive tasks.

The lack of transfer effects in children is puzzling, as previous studies with populations known to have episodic memory deficits, such as older adults [31,35,37] or people with depression [39], have shown transfer from ESI to subsequent cognitive tasks involving episodic thinking. For example, Madore et al. [37] studied ESI in older adults, whose ability to recall memories from the past typically shows some decline due to impairments in the prefrontal cortex and middle-temporal lobes [4]. In this study, older adults, similar to their younger counterparts, showed a benefit on a divergent thinking task (AUT) after an ESI interview, suggesting that enhancing the use of episodic memory with the ESI procedure produces benefits on tasks that also involve episodic memory, even in individuals with weaker memory function.

The failure to observe transfer effects in the Tanguay et al. study [38] may be due to the use of a future-oriented ESI interview rather than the standard ESI procedure, which focuses on retrieving past information. Imagining future events may be more cognitively demanding for children (see [40]) and/or results in lower levels of episodic retrieval. Therefore, manipulations that increase episodic engagement in the ESI interview by using a standard retrospective ESI interview may be crucial when adapting ESI for younger participants. In fact, previous studies have demonstrated improvements in later creativity tasks when similar processes are engaged during ESI. Thus, for example, Zhang et al. [41] found better performance in a creativity task consisting of a poetry composition only when a poetry-related video was provided for the standard ESI interview but not with a non-poetry video. Hence, the similarity between the episodic processes engaged during the ESI interview and the later creativity task seemed critical for transfer.

Therefore, the present study aims to investigate whether enhancing children’s episodic recall from a video using the standard ESI procedure from Madore et al. [31], can improve their performance in a subsequent divergent thinking task that is assumed to involve episodic thinking (i.e., Alternative Uses Test). In this sense, the type of material used when conducting ESI might also play a fundamental role in the effectiveness of this induction in children. Thus, Experiment 1 introduces a novel form of ESI designed to facilitate episodic memory in children by using child-friendly videos. These videos are clips from an animated TV show following a story line and might be more familiar and engaging within children population. The materials used to elicit episodic recall plays a fundamental role in studies where participants learn or recall details from a video [42–46] and, specifically, memory retention tends to improve with materials that promote engagement [46]. In addition, children possess familiarity with the structural, linguistic, and narrative elements of animated TV shows. In this sense, developmental studies have found that children’s episodic memory improves when they are asked to remember things they are experts at [47]. Thus, when they can rely on their prior knowledge (i.e., schema knowledge) to provide conceptual regularities to integrate other events [48,49], they show better memories. Hence, Experiment 1 introduces a novel form of ESI designed to facilitate episodic memory engagement in children by using child-friendly videos (e.g., clips from an animated TV show) as the basis for the ESI interview, while Experiment 2 adopts the standard ESI procedure, using the same videos as in Madore et al. [36,37] which originally were non-adapted to children.

## Experiment 1

In Experiment 1, we examined whether second graders (seven to eight years old), fifth graders (ten to eleven years old), and young adults differ in their episodic recall during ESI and whether they transfer its benefits to other tasks (i.e., creativity tasks) when using adapted audiovisual materials, such as short clips from TV cartoons. Based on previous studies [38], we expected the ESI interview to elicit more episodic memories than the control interview across all age groups. Additionally, we anticipated an age-related increase in the number of specific episodic details recalled from the video (as seen in [25]). However, we hypothesized that this difference might be reduced by the child-friendly nature of the audiovisual material, which may enhance children’s recall of episodic details, thereby minimizing or even eliminating age-related differences.

Furthermore, we tested for age differences in episodic memory by examining the effect of ESI on two creativity tasks: a divergent thinking task (i.e., the ability to generate numerous solutions in response to a single problem) and a convergent thinking task (i.e., the ability to generate the best single solution to a given problem) [50]. Previous studies with adults have shown that ESI has an effect on a divergent thinking task (i.e., Alternative Uses Task, AUT) but not on a convergent thinking task (i.e., Remote Associates Test, RAT) [36]. This is likely because convergent thinking tasks rely less on episodic retrieval processes and therefore benefit less from ESI (see [51]).

Based on these findings, we expected to observe an ESI effect on the AUT but not on the RAT. Although Tanguay et al. [38] found no benefits of future-oriented ESI on other cognitive tasks in young children (six to seven years old), we expected that adapting the standard ESI procedure to better support episodic memory in our child sample would lead to an increase on the AUT number of ideas (fluency and appropriate uses) and variety of ideas (flexibility and categories of appropriate uses) in both young and older children, as well as young adults. However, no effect of ESI was expected on originality or elaboration.

### Method

#### Participants.

The sample size was estimated based on the study by Madore et al. [37] They performed a power analysis (G*Power 3 [52]) and determined that 24 participants are needed for each between-condition (age group) to observe a medium effect size (d = .60, power > .80, two-tails) for a within-subject variable (type of induction) and to observe a large effect (d = .80, power > .80, one-tail) for the between-group variable (age group). A total sample of 72 participants from three age groups was collected: 24 of them were studying their second grade of primary school (M_age_ = 7.33; SD = 0.48), 24 of them were studying their fifth grade of primary school (M_age_ = 10.4; SD = 0.5) and 24 of them were young adults (M_age_ = 21.1, SD = 1.86).

The majority of the participants were volunteers recruited through social media via advertisements seeking individuals for a study on memory. To minimize potential bias from participant’s guessing the study’s hypothesis, we asked parents not to disclose the study’s purpose to their children, instead telling them they would be completing tasks involving watching a video and answering questions about it. Regarding adult participants, some received 0.3 course credits for their participation in psychology course. At the end of the experiment, they were asked if they could guess the study’s hypothesis, but none of them were able to do so.

Before completing data collection, 12 young adults were removed from the sample and replaced due to a mistake in the counterbalance procedure. Participants were also balanced in terms of gender (36 men and 36 women) and Spanish was the first language for all of them. All participants (parents in the case of minors) gave written or online informed consent in accordance with the guidelines approved by the ethics committee at the University of Granada (code: 917/CEIH/2019).

Additionally, we included some control measures of working memory (Dot-Counting; [53] problem-solving (Matrices subtest, K-BIT; Kaufman, 1997), and vocabulary (vocabulary subtest, K-BIT [54]; and verbal fluency task [55]) to test developmental differences within the sample. Results are shown in Table 1.

**Table 1:** Average scores (SD in brackets) in the control tasks separated by age group.

Grade	Dot Counting (SPAN)	Matrices	Vocabulary	Verbal Fluency
Second graders (2nd)	4.13 (1.12)	25.41 (4)	35.25 (3.8)	–
Fifth graders (5th)	5.00 (0.78)	31.92 (5.34)	39.33 (3.14)	–
Young Adults (YA)	5.46 (0.83)	38.13 (3.84)	–	75.42 (13.77)
Group differences (*p* < .05)	2nd < 5th = YA	2nd < 5th < YA	2nd < 5th	–

### Materials

1 **Audiovisual material**

The audiovisual materials were children-adapted. We selected two short clips (3:14 minutes each) from an Andalusian TV animation series called “Bandolero” [56]. Each video featured a structured story with various animated characters. The language and content of the story were age-appropriate, similar to other children’s cartoons. Both clips were matched in duration, number of characters, and type of animation, but to avoid practice effect the two clips belonged to different episodes and the characters differ for each episode.

Moreover, the two included humorous, entertaining moments (e.g., a baby soiling his diaper in Video 1 or a child falling off a wild pig in Video 2) as well as some emotionally charged negative moments (e.g., a mother abandoning her child in Video 1 or a soldier burning down a house in Video 2) in order to increase attentional engagement (Liu et al., 2013). Furthermore, we chose a series broadcasted on 2001 in an autonomic TV channel, to minimize the likelihood that children had previously seen it and reduce the probability that recently viewed content could lead to a ceiling effect in recall.

Since young adults in the study might have seen the series during their childhood, they were asked at the end of the experiment whether they recognized the clips. While most of the young adults (83.4%) reported having seen the series during their childhood, they all reported no recollection of any specific details from the videos.

2 **ESI/Control interview**

During the *Episodic Specificity Induction (ESI)* interview, the experimenter asked the participants to close their eyes and to imagine the surroundings of the video in order to tell everything they could remember about the places as detailed as possible. After the participant told the experimenter what they could remember, questions about concrete details were provided following the protocol of Madore et al. [31] (i.e., “do you remember the color of the cave that you had mentioned?”, “were there another place in the video?”…). After that, the same procedure was followed regarding the characters in the video and their actions.

The *control* interview (impressions interview) consisted of a pool of 20 questions from Madore et al. [31] in which the experimenter asked the children about their impressions and general opinions on the clip that they had seen (for example, “did you enjoy the video?”, “who was your favorite character and why?” …).

The only differences between our protocol and the original Madore et al. [31] protocol were the translation into Spanish and slight modification to align with the new video. See S1 Appendix in S1 File for a detailed description of the ESI and the Control interview protocol.

3 **Alternative uses test** (**AUT**) [57,58]

This test measures divergent thinking, this is, our capacity to generate the greater number of alternative responses from a single idea (participants were asked to generate as many uncommon uses as possible for a given object). As our participants went through two different conditions, we created 2 sets of 3 items. We selected these items based on the items with high reliability and validity in the infant population [59], we chose for set A “periódico” (newspaper), “botón” (button), and “neumático” (car tire) (frequency = 38.49), and for set B “cuchillo” (knife), “zapato” (shoe) and “llave” (key) (frequency = 16.99), with no significant differences in relative frequencies taken from Guasch et al. (2013), Welch’s *t*(2.04)=−0.682; *p* = .564.

4 **Remote associates test** (**RAT**) [60]

This test measures convergent thinking, defined as the ability to associate ideas that apparently seem to be unconnected or remote to generate a single correct solution [50]. For the two groups of children (second graders and fifth graders) we created 2 sets of 20 items matched according to their mean of correct responses (set 1: M = .6, SD = 0.15; set 2: M = .63, SD = 0.17) and item-test correlation (set 1: M = 0.38, SD = 0.14; set 2: M = 0.39; SD = 0.11) in infant population, extracted from Peláez-Alfonso et al. [61]. A pilot study with 10 young adult participants showed a ceiling effect when using these same items (most of them obtained more than 90% of hits). Hence, we generated 2 new sets of items from Peláez-Alfonso et al. [61] with higher difficulty for the adult sample. These two sets were matched in the mean of correct responses (set 1: M = 0.6, SD = 0.14; set 2: M = 0.6, SD = 0.16) and item-test correlation (set 1: M = 0.23; SD = 0.1; set 2: M = 0.26; SD = 0.13), all *p*s > .05. Items were divided into 3 different blocks: semantic (i.e., rice, rings, white – wedding), compound-words (i.e., biography, mobile, pilot – auto) and two-word expressions (i.e., reproductive, circulatory, electrical – system) (selected items in Spanish specified in S2 Appendix in S1 File).

#### Procedure.

Seven participants conducted this experiment in March of 2020, beginning on the 1^st^, at a laboratory of the Research Center Mind, Brain and Behavior (CIMCYC, Granada). The experiment had two sessions (with approximately 1 week between them). To ensure consistency, the same experimenter conducted all sessions in the same room for all participants. Due to the Covid-19 emergency, the experiment was adapted to an online format, with data collection concluding on May 15, 2020. The design remained unchanged with the only difference that the tasks were conducted via *Google Hangouts*. Since environmental context might influence the ability to generate creative ideas [62,63], participants were asked to remain in the same room for both sessions, and the parents were instructed not to intervene in any of the tasks if they were present. The same pattern of results was observed in the in-person and online participants (see S3 Appendix in S1 File). Informed consent was obtained from parents before the experiment.

During each session, a video was first presented, and the experimenter asked the participants to pay full attention for a later test. The two videos were counterbalanced between sessions. Once they had seen the video, participants would engage in *the Episodic Specificity Induction* (ESI) interview (M_min_ = 6.9, SD = 1.46) or in the *Control* interview (M_min_ = 5.03, SD = 1.02) following the same protocols of Madore et al. (2014). Inductions were also counterbalanced between sessions.

Then, counterbalanced across participants, the Alternative Uses Test (AUT) or Remote Associates Task (RAT) were carried out. Regarding the Alternative Uses Test (AUT), participants were instructed to generate out loud as many uses as they could for a daily life object (instruction on fluency). They were allowed to generate the common uses but they were encouraged to generate unusual uses different than the common one (instruction on creativity) (i.e., “brick” is typically used for constructions but you could use a brick as a pillow, as a nutcracker or to play with many of them and to make a “domino effect”). When the experimenter was sure that the participant understood the instructions, the first object was presented and participants had 2 minutes to generate as many alternative uses as possible (see S4 Appendix in S1 File). Since participants were children completing the study online, some occasionally refrained from responding due to shyness or difficulty understanding the task. To ensure active engagement and idea generation, the experimenter was allowed to provide encouraging prompts such as “any possible idea is valid”, “you can let your imagination run free and see what comes to your mind”, “imagine you are bored with this object, what else could you use it for?” To prevent potential bias, any prompts given in one condition were consistently provided in the other, ensuring that their influence remained uniform across conditions.

The Remote Associates Task (RAT) was conducted through the program E-Prime 2.0. [64], using the screen-share option for the online version. Instructions appeared on the screen, and they were read out loud to make sure participants would understand. During this task, triads of three words (i.e., lack, pocket, and ozone) were presented visually accompanied by an audio track to participants and they had 20 seconds to generate a word that was related to these three concepts (i.e., hole). Additionally, following AUT and RAT, participants conducted the control tasks.

#### Design.

The experiment followed a 3 (age group: second graders, fifth graders, and young adults) x 2 (type of interview: ESI and control) mixed factorial design with age group as a between-group variable and type of interview as a within-subject variable (see Table 2).

**Table 2:** Factorial matrix.

Age Group	Interview Type (within-subject, order counterbalanced)	
	ESI	Control
Second graders (n = 24)	${\overset{―}{Y}}_{second,\;ESI}$	${\overset{―}{Y}}_{second,\;Control}$
Fifth graders (n = 24)	${\overset{―}{Y}}_{fifth,\;ESI}$	${\overset{―}{Y}}_{fifth,\;Control}$
Young adults (n = 24)	${\overset{―}{Y}}_{adults,\;ESI}$	${\overset{―}{Y}}_{adults,\;Control}$

#### Scoring.

Episodicity of Responses during ESI and Control Interviews was evaluated by two independent raters following a two steps procedure: first responses were segmented into informational bits or details ([65]; see S5 Appendix in S1 File for a complete description), second, following Purkart et al. [66], each detail was categorized into:

1. Episodic specific details: Concrete pieces of information related to the place, people, and actions, retrieved from the video (i.e., “there was a girl with a pink T-shirt”, “the man was sleeping at the beginning”, “the cave was grey”…).
2. General Details: Sematic information (i.e., “kitchens usually have a sink”), opinions and impressions (i.e., I think it is awful she did that), or hypothesis (i.e., “I imagine they were using the gloves to clean).
3. Additionally, we included a third category called “Episodic external details” in order to consider pieces of information not related to the video but that could elicit episodic thinking, like autobiographical memories, details from the video watched in the first session one week after that, or future imagining.

As the two raters showed high intraclass correlation (IC) within nine participants, randomly chosen from the sample pool, in the number of episodic specific details (IC = .97) and general details (IC = .92), the first rater continued coding the remaining participants.

Responses from the Alternative Uses Test were rated by two independent raters who evaluated six divergent thinking dimensions as dependent variables (fluency, flexibility, number of appropriate uses, categories of appropriate uses, originality, and elaboration) (see Madore et al., 2016 for a similar procedure).

*Fluency* (or *total number of uses* according to Madore et al. [36]): number of uses that the participant gives to the three objects on each AUT set. Although some authors recommend to eliminate uses that are considered common (i.e., I would use a knife to cut food) or that could be applied to any object (i.e., I would buy, keep, or borrow that object) [67]), we did not exclude any use to be consistent with Madore et al. [38]. However, results removing common uses did not significantly affect results (see S6 Appendix in S1 File).

*Flexibility* (or *categories of uses* according to Madore et al. [36]): number of categories in which the uses given by the participant can be classified, averaged per object prompt (based on [68]). This dimension reflects the ability to give different types of answers and shift from one category to another. Both raters used categories from Goldenberg and Wiley [69], adding new categories when the uses did not match any of the existing ones.

*Number of appropriate uses* was scored by summing all the uses that are considered feasible (encoded as 1). For example, an unrealistic responses such as “I would use a shoe to fly” would be scored as 0, whereas plausible responses like “I would use a shoe as a flower pot” would be scored as 1.

*Categories of appropriate uses* measures flexibility of the uses that were considered appropriate, that is to say, the number of categories (averaged per object prompt) in which we can classify the uses that were considered appropriate or feasible according to the raters.

*Originality* is the dimension that identifies how novel or uncommon are the alternative uses given to the objects on a scale from 1 (not novel at all) to 5 (very novel). The final originality score was calculated by averaging originality participant’s scores (i.e., Sum of originality scores/total number of uses in each condition).

*Elaboration* consists of how detailed is the description of an idea, and it was assessed by using a scale from 1–3, being 1 not detailed (i.e., I would use a shoe as door stop), 2 a bit detailed (i.e., I would use a shoe as a door stop to prevent a door slamming) and 3 very detailed (i.e., I would use a shoe as a door stop to prevent a door slamming in case it is a windy day). The final elaboration score was calculated by averaging elaboration participant’s score (i.e., Sum of elaboration scores/total number of uses in each condition) following ESI or control condition.

Results showed high intraclass correlation (IC) (consistency analysis) for every dimension: flexibility (IC = .93), appropriate uses (IC = .97), categories of appropriate uses (IC = .91), originality (IC = .83), elaboration (IC = .79).

For RAT correction, we calculated the percentage of hits following a strict criterion (i.e., participant response corresponded to the more frequent response in Peláez-Alfonso et al. [61]), and following a lenient criterion (i.e., participant response was correct but it did not correspond to the more frequent response in the normative study, for example, for the triad “nature, peaceful, grass” the response “forest” would not be considered correct following the strict criterion but it would be under the lenient criterion).

### Results

#### Interview.

Table 3 reports the raw number of details generated during ESI and control interviews across the three age groups. As there were almost no external episodic details during ESI (M = 0.042, SD = 0.201) or control (M = 0.819, SD = 0.924), they were not analyzed separately but were instead included when counting for the total number of details.

**Table 3:** Averages (SDs in brackets) of episodic and general details by group and type of interview.

Group	Episodic Specific Details		General Details		Total number of details
	ESI	Control	ESI	Control	
Second graders	26.13 (6.32)	4.63 (2.72)	3.04 (2.26)	13.21 (4.8)	47. 8 (10.6)
Fifth graders	31.96 (6.73)	5 (2.45)	3.29 (2.55)	15.42 (5.85)	56.6 (11.6)
Young Adults	39.04 (9.03)	3.25 (2.03)	4.79 (3.11)	27.58 (5.68)	75.5 (11.4)

Since age group was treated as a categorical variable in this study, a 3 (age group: second graders, fifth graders vs. young adults) x 2 (type of interview: ESI vs. control) ANOVA on the **raw number of episodic specific details** was carried out. As expected, results revealed a higher number of episodic specific details during ESI (M = 32.4; SD = 9.07) compared to the control interview (M = 4.29; SD = 2.50), *F*(1,69) =1017.5; *p* < .001; η^2^_p_ = 0.936. In addition, there was a significant main effect of age, *F*(2,69) =11.9, *p* < .001; η^2^_p_ = 0.257, revealing a lower number of episodic specific details in second graders (M = 26.13; SD = 6.32) compared to fifth graders children (M = 31.96; SD = 6.73), *t*(69)=−2.63; *p*_*tukey*_ < .028, and adul*t*s (M = 39.04; SD = 9.03), *t*(69)=−4.88; *p*_*tukey*_ < .001. Moreover, the in*t*eraction type of induction x age group reached statistical significance, *F*(2,69) =22.4; *p* < .001; η^2^_p_ = 0.393. This interaction showed an age effect in ESI where young adults provided a higher raw number of episodic specific details compared to children (young adults vs second graders, *t*(69)=−6.001; *p*_*tukey*_ < .001; young adults vs fifth graders, *t*(69)=−3.291; *p*_*tukey*_ = .019, but there were no differences between children, *t*(69)=−2.710; *p*_*tukey*_ = .086. However, this age effect was not observed in the con*t*rol condition (all *p*s > .05). See Table 2.

Overall, the number of **total details** produced by the participants increased with age, F(2,69)= 39.2; *p* < .001; η^2^_p_ = 0.532, indicating a larger fluency in the production of details for adults compared to fifth graders, *t*(69)=−5.95; *p*_*tukey*_ < .001; and followed by second graders *t*(69)=−2.71; *p*_*tukey*_ = .023 (see Table 3). Since our primary in*t*erest was to examine the degree of episodicity in each condition while controlling for differences in overall verbal production, we calculated the **proportion of episodic specific details** within each condition by dividing the number of episodic specific details by the total number of details produced in that same condition (See Table 3).

A 3 (age group: second graders, fifth graders vs. young adults) x 2 (type of interview: ESI vs. control) ANOVA on the proportion of episodic specific details per condition revealed a main effect of type of interview, *F*(1,69) = 3013.95; *p* < .001; η^2^_p_ = 0.978, with a higher proportion of episodic specific details in the ESI interview (M = 0.9; SD = 0.07), compared to the control interview (M = 0.19; SD = 0.11),. A main effect of age group also emerged, *F*(2,69) =10.9; *p* < .001; η^2^_p_ = 0.239, with second graders, *t*(69)= 3.85; *p*_*tukey*_ < .001; and fif*t*h graders, *t*(69)= 4.2; *p*_*tukey*_ < .001, showing a significantly higher propor*t*ion of episodic specific details compared to young adults. However, no difference was found between second and fifth graders, *t*(69)= 0.36; *p*_*tukey*_ = .833.

Furthermore, a significant type of interview x age group interaction was observed, *F*(2,69) =9.77; *p* < .001; η^2^_p_ = 0.221. Specifically, there were no significant age differences in the proportion of episodic specific details during the ESI interview (adults vs. second graders, *t*(69)= 1.1; *p*_*tukey*_ = .88; adults vs. fifth graders, *t*(69)= 0.16; *p*_*tukey*_ = 1; second graders vs. fifth graders *t*(69)= 0.94; *p*_*tukey*_ = .935). However, during the control interview, adults produced a significantly lower proportion of episodic specific details (M = 0.1; SD = 0.06) compared to both second graders (M = 0.23; SD = 0.11), *t*(69)= 5.17; *p*_*tukey*_ < .001, and fifth graders (M = 0.24; SD = 0.01), *t*(69)= 4.89; *p*_*tukey*_ < .001. No differences be*t*ween second and fifth graders were observed in the control interview, *t*(69)= −0.28; *p*_*tukey*_ = 1 (see Table 4).

**Table 4:** Averages (SDs in brackets) of the proportion of episodic specific and general details over total produced details per condition.

Group	Proportion of Episodic Specific Details		Proportion of General Details	
	ESI	Control	ESI	Control
Second graders	0.89 (0.08)	0.24 (0.01)	0.11 (0.08)	0.73 (0.06)
Fifth graders	0.91 (0.06)	0.23 (0.11)	0.09 (0.06)	0.72 (0.13)
Young Adults	0.89 (0.08)	0.1 (0.06)	0.11 (0.08)	0.87 (0.06)

#### Divergent thinking: Alternative uses test (AUT).

For the Alternative Uses Test (AUT), we conducted separate 3 (age group: second graders, fifth graders, young adults) x 2 (type of interview type: ESI vs. control) ANOVAs for each of the AUT dimensions individually. Specifically, we included fluency, flexibility, appropriate uses, categories of appropriate uses, originality and elaboration as AUT dimensions replicating Madore et al. (2016) dependent variables.

Results revealed a main effect of type of interview on fluency, *F*(1,69)=7.62, *p* = .007, η^2^_p_ = 0.099, appropriate uses, *F*(1,69)=10.255, *p* = .002, η^2^_p_ = 0.129, flexibility, *F*(1,69)=5.51, *p* = .017, η^2^_p_ = 0.08, categories of appropriate uses, *F*(1,69)=5.191, *p* = .026, η^2^_p_ = 0.07, and elaboration, *F*(1,69)=4.39, *p* = .04, η^2^_p_ = 0.06. Surprisingly, participants generated a significantly higher number of ideas (fluency and appropriate uses) and categories of ideas (flexibility and categories of appropriate ideas) in the control condition compared to ESI. On the contrary, ideas were less elaborated in the control condition compared to ESI (see Table 5). Conversely, the main effect of the type of interview for originality did not reach significance, *F*(1,69)=1.71, *p* = .195, η^2^_p_ = 0.024.

**Table 5:** Means (M) and standard deviations (SD) in alternative uses test (AUT) dimensions.

Age Group	ESI				Control			
	Second Grader	Fifth Grader	Young Adults	Total	Second Graders	Fifth Graders	Young Adults	Total Sample
Fluency	16.5 (7.44)	24 (10.2)	27.2 (7.61)	22.6 (9.50)	18.8 (10.3)	26.8 (11.5)	28.5 (6.75)	24.7 (10.5)
Flexibility	3.44 (1.11)	4.5 (1.29)	5.24 (1.24)	4.39 (1.41)	4.01 (1.72)	5.01 (1.58)	5.33 (1.20)	4.78 (1.60)
AU	14.3 (5.28)	21.4 (7.45)	25.5 (7.13)	20.4 (8.07)	15.9 (7.19)	24.1 (9.42)	27.5 (6.45)	22.5 (9.12)
CAU	3.24 (1.04)	4.31 (1.26)	4.97 (1.13)	4.17 (1.34)	3.67 (1.52)	4.67 (1.39)	5.18 (1.12)	4.51 (1.47)
Originality	2.68 (0.73)	2.79 (0.53)	3.07 (0.33)	2.85 (0.57)	2.79 (0.63)	2.9 (0.45)	3.1 (0.41)	2.93 (0.52)
Elaboration	1.41 (0.46)	1.44 (0.31)	1.63 (0.32)	1.49 (0.38)	1.32 (0.37)	1.32 (0.27)	1.64 (0.33)	1.43 (0.35)

Note: *AU: Appropriate Uses, CAU: Categories of Appropriate Uses.

On the other hand, the type of interview x age group interactions were not significant in any of the AUT dimension: fluency, *F*(2,69)=0.329, *p* = .721, η^2^_p_ = 0.009, appropriate uses, *F*(2,69)=0.251, *p* = .778, η^2^_p_ = 0.007, flexibility, *F*(2,69)=0.85, *p* = .432, η^2^_p_ = 0.024, categories of appropriate uses, *F*(2,69)=0.209, *p* = .812, η^2^_p_ = 0.006, originality, *F*(2,69)=0.138, *p* = .872, η^2^_p_ = 0.004, and elaboration, *F*(2,69)=1.62, *p* = .205, η^2^_p_ = 0.045.

Finally, there was a significant main effect of age in all AUT dimensions: fluency, *F*(2,69)=9.25, *p* < .001, η^2^_p_ = 0.211, appropriate uses, *F*(2,69)=18.1, *p* < .001, η^2^_p_ = 0.344, flexibility, *F*(2,69)=10.6, *p* < .001, η^2^_p_ = 0.235, categories of appropriate uses, *F*(2,69)=13.7, *p* < .001, η^2^_p_ = 0.284, elaboration, *F*(2,69)=5.48, *p* = .006, η^2^_p_ = 0.137, originality, *F*(2,69)=3.74, *p* = .029, η^2^_p_ = 0.098. Specifically, young adults scored higher compared to second graders (all *p*s < .05) in all AUT dimensions. Moreover, in the elaboration dimension, young adults showed significantly higher scores compared to fifth graders (*p*s < .05) and in the dimensions of fluency, flexibility, appropriate uses and categories of appropriate uses fifth graders children showed significantly higher scores compared to second graders (*p*s < .05) (relevant results from Experiment 1 and 2 are summarized in S7 Appendix in S1 File).

Finally, it is important to note that including the counterbalancing order of the interviews as a between subjects variable in the ANOVA did not alter the results. The main effect of order and its interactions with age or type of interview was non-significant for all AUT dimensions (all *p*s> .05).

#### Convergent thinking: Remote associates task (RAT).

Since we adapted the difficulty of RAT to the children and adults’ groups for analyses, we calculated the proportion of RAT correct responses (we used both strict and lenient criteria as mentioned above). We performed two ANOVAs 3 (group: young adult, fifth graders children, second graders children) x 2 (type of interview: episodic vs. control) on RAT scores for the two types of criteria (strict and lenient) as dependent variables.

Results revealed that the main effects of the type of interview were not significant, strict, *F*(1,69)=0.990, *p* = .323, η^2^_p_ = 0.014, and lenient criterion, *F*(1,69)=0.427, *p* = .511, η^2^_p_ = 0.006 (see Table 6). The interaction type of interview x age was also non-significant, strict, *F*(2,69)=2.221, *p* = .116, η^2^_p_ = 0.060, and lenient criterion, *F*(2,69)=1.14, *p* = .326, η^2^_p_ = 0.032.

**Table 6:** Means (M) and standard deviations (SD) of the proportion of hits on RAT.

Group	Strict Criterion		Lenient Criterion	
	ESI	Control	ESI	Control
Second Graders	0.46 (0.11)	0.54 (0.11)	0.48 (0.1)	0.53 (0.1)
Fifth Graders	0.51 (0.09)	0.49 (0.09)	0.51 (0.07)	0.49 (0.07)
Young Adults	0.5 (0.09)	0.5 (0.09)	0.5 (0.1)	0.5 (0.1)

### Discussion

The first aim of Experiment 1 was to analyze developmental differences in the amount of episodic recall during ESI and control interviews. The results indicated that the three age groups provided a significantly different number of episodic specific details during the ESI compared to the control interview. Interestingly, the young adults were better able to adapt to the demands of the task than the children and they provided a significantly higher number of episodic details in this condition. This pattern is consistent with the results reported by Coughlin et al. [25,26] which indicate developmental differences in the number of episodic details recalled by children of different ages. Similarly, Benear et al. [70] found that adults recalled significantly higher number of episodic specific details from a cartoon TV show compared to 4- to 7-year-old children.

However, we also observed that this pattern might be due to the higher fluency of adults, who produced many more details than second and fifth graders. Indeed, a previous study demonstrated that the mean number of words provided in past and future narratives differed significantly between seven-, nine-year-old children and adults [26]. When we analyzed the relative proportion of episodic details from the video (i.e., the number of episodic specific details divided by the total number of details within each interview), the differences between children and adults during ESI were reduced, and the effect of age was no longer significant in this condition. This finding suggests that, although children recalled fewer episodic details in absolute terms, when controlling overall verbal output, the proportion of episodic details was comparable between children and young adults.

Conversely, during the control interview, where participants were asked to generate general thoughts and opinions about the video, children produced a significantly higher proportion of episodic details compared to adults. This finding may be explained by the fact that adults recalled a greater proportion of general details compared to children. Thus, it is possible that when the task specifically requires providing general impressions of a video, adults are better able than children to adapt to the requirement of the task.

Furthermore, all participants, regardless of age, showed a significantly higher degree of episodicity in the ESI condition compared to the control condition, consistent with findings by Tanguay et al. [38] who observed similar effects when applying future-oriented ESI in children.

Given that the ESI procedure was successful in inducing higher levels of episodicity in second graders, fifth graders and young adults, the second question was whether participants would benefit from ESI when performing the subsequent divergent (AUT) and convergent (RAT) thinking tasks. In this regard, we found no significant effect of ESI on the convergent thinking task (RAT). These results were expected, given that a previous study by Madore et al. [36] found no significant transfer effect of induction on RAT. Convergent thinking tasks, such as the RAT, are more related to semantic processes than episodic retrieval and may therefore not benefit as much from an episodic retrieval orientation [51].

However, the results for the AUT showed an unexpected pattern. Across all three age groups, participants provided a significantly greater number of ideas and categories of ideas after the control condition than after the ESI. On the contrary, the elaboration score was significantly higher after the ESI than after the control interview. These results did not match our expectations, as based on the results of Madore et al. [36,37], we expected a higher number of uses following ESI than the control condition and no effect of induction on elaboration. One possible explanation is the inverse relationship between fluency and elaboration in time-limited AUT tasks. In this line with this, Arabian et al. [71] found that participants following ESI obtained a higher score on elaboration in a 1-min AUT, but no effect on fluency compared to control condition. Thus, it is possible that participants following ESI elaborated their ideas more at the cost of a significantly lower number of ideas while the opposite pattern may have occurred in the control condition.

Alternative explanations should consider the cognitive mechanisms differentially engaged by the control interview and ESI and their potential impact on AUT idea generation. In particular, inhibitory control may play a crucial role on this. Several studies have suggested that in divergent thinking tasks, where participants must generate open-ended responses, a reduction in cognitive control mechanisms can actually facilitate idea generation [72,73]. In this context, ESI is a goal-directed interview that requires participants to focus their attention narrowly on retrieving as many specific details as possible from the video. As a result, it is plausible that ESI increases inhibitory control, which, in turn, reduces idea fluency in the subsequent AUT compared to the control interview. Supporting this hypothesis, research by Chrysikou & Thompson-Schill [72] indicates that deliberate retrieval processes, such as those required during ESI, engage greater cognitive control and prefrontal activation compared to more spontaneous memory retrieval, as might occur during control interview, where participants provide broad general though without retrieving specific details. However, this inhibitory hypothesis cannot account for previous results that observed more appropriate uses in divergent thinking after the ESI condition [36,37].

Finally, it also is possible that the provided materials (cartoons) could elicit a positive mood which differentially affects creativity depending on the interview type. Several studies found that inducing positive emotional states might enhance subsequent idea generation in creativity tasks [74–78]. For instance, Kaufman & Vosburg [76] found that participants exposed to positive mood-inducing film clips increased early-stage idea production when generating alternative uses for a shoe. In line with this, it is possible that generating general thoughts about the video during the control interview, particularly for child participants, may have fostered a more positive emotional state. Unlike ESI, the control interview imposed no pressure to recall details accurately, allowing participants to freely discuss the entertaining cartoons they had just watched. In contrast, the ESI condition required them to focus on retrieving specific details, a more difficult task that may have been less influenced by the emotional valence of the video itself. Furthermore, as suggested by Tanguay et al. [38], the ESI condition required, on average, a longer completion time, which may have led to greater cognitive fatigue compared to the control impressions interview. This depletion of the cognitive resources could have further attenuated potential benefits in the subsequent AUT.In summary, using children-adapted materials (TV cartoons), we found that, like adults, children recalled more episodic details after an ESI interview than in a control condition. Additionally, following ESI, they generated more elaborate alternative uses in the subsequent AUT task, although fluency, flexibility, appropriate uses and categories of appropriate uses showed the opposite pattern. The relative advantage in elaboration for children could be explained by the use of children-adapted materials in the present experiment. As noted above, using highly engaging videos may increase memory retention [46] and minimize developmental differences in recall. However, the increase in fluency and flexibility observed following the control interview highlights the need to consider additional factors that may influence the transfer effects in both ESI and control interviews.

## Experiment 2

In Experiment 2, we aimed to test whether the effect of ESI on children’s episodic processing and its transfer to the AUT is modified when standard ESI materials from Madore et al. [31,36,37] are used. These standard materials consisted of videos depicting adults performing activities in a kitchen in a random order (i.e., putting flowers in a pot, placing a saw over the refrigerator…). Although the videos depicted easily understandable actions for children, unlike the cartoon clips in Experiment 1, they lacked a clear narrative and were likely less engaging. Therefore, we used them first to replicate and extend Madore et al. [31,36,37] results to other age population and, second, to assess if the type of video modulated episodic memory processes in children. This question is particularly relevant given that, aside from Tanguay et al. [38], who used a more challenging future-oriented version of ESI, to our knowledge, no previous studies have implemented the standard ESI procedure with children.

An additional difference between the procedure of Experiments 1 and 2 was that, at the end of the divergent thinking task, participants were asked to indicate whether each creative use produced was old (they recalled this use from memory) or new (they thought of this creative use for the first time during the experiment). This measure was introduced by Madore et al. [31,36,37] and allows us to disentangle ideas that come directly from episodic memory (‘old’) from those that arise from the recombination of semantic information and imagery (‘new’) [36].

Thus, the questions in Experiment 2 were (1) whether changing the video material to less entertaining videos would modulate children’s ESI performance; (2) whether children would benefit from ESI in a subsequent divergent thinking task (AUT) when using these non-adapted video materials; and (3) whether the old/new measure could also be used with children to disentangle the source of their responses in the creative task.

To this end, we selected a sample of fifth grade children of the same age and conditions as the older groups of children in Experiment 1. This age group was chosen over the younger ages also present in Experiment 1 in order to avoid possible floor effects for the younger children. Overall, and similar to Experiment 1, we expected that children would produce more episodic details in the ESI than in the control interview, although the difference might be smaller than in Experiment 1. In addition, we expected that difficulty of episodic encoding and retrieval of the studied material might result in no effect of ESI on the subsequent divergent thinking task (AUT). If this were the case, our results would be similar to those of Tanguay et al. [38]. We also wanted to investigate whether participants provided more ideas labelled as ‘old’ or ‘new’ after ESI (compared to the control condition), as Madore et al. [37] found a significant increase in both types of ideas after ESI, but this increase was greater for ‘old’ ideas.

### Methods

#### Participants.

The sample size was calculated using G*Power analysis to determine the number of participants to observe a medium effect size (d = .60, power > .80, two-tailed) based on the power analysis of Madore et al. [36]. As a result, 24 volunteer children (9 boys and 15 girls) in the fifth year of primary school (Mage = 10.2; SD = 0.415) were selected to participate in the experiment. Participants were recruited through online advertisements via social media (e.g., whatsapp, instagram posts, twitter) as in Experiment 1. Prior to participation, parents gave written informed consent, which was handled according to guidelines approved by the Ethics Committee of the University of Granada (code: 917/CEIH/2019). In addition, they were questioned about possible illnesses or developmental disorders in the children. From this, we found that two children were diagnosed with autism spectrum disorder, but since any intellectual disability was reported, they were not excluded from the sample. However, one of the participants with psychopharmacological treatment was removed from the sample.

#### Materials.

As in Experiment 1, participants performed the same version of Alternative Uses Test and induction protocol from Madore et al. [31]. For the ESI and Control two short videos (1:54 minutes) from Madore et al. [31,36,37] studies were used. These videos depicted a man and a woman performing various everyday action in the kitchen in a random order. Since the characters spoke occasionally, the videos were subbed into Spanish to ensure comprehension for all participants.

#### Procedure.

The procedure was similar to Experiment 1 but this version was conducted in the laboratory rather than online. This experiment began on February 6, 2023, and concluded on March 31, 2023. Participants watched one of the two videos from Madore et al. [31], after which they conducted either ESI (M_min_ = 5.29; SD = 0.81) or control interview (M_min_ = 4.56; SD = 0.66) (See S1 Appendix in S1 File). Following the interview (ESI or control, depending on the counterbalancing condition), the AUT was administered. The AUT instructions were slightly modified to ask participants to generate only alternative uses for the objects. This modification was made to enhance clarity and ensure a better understanding of the task, while maintaining the core focus. Additionally, it helped align the instructions more closely to those used by Madore et al. [36] (see S4 Appendix in S1 File).

Moreover, following the procedure of Madore et al. [36] with adults, the experimenter did not provide any input during the AUT as the experiment was not online. Unlike in Experiment 1, at the end of the second session, participants had to label their ideas as ‘old’ or ‘new’. Ideas were classified as new if they came to mind for the first time during the experiment (i.e., “I just thought of using a key to cut a cake”) and as old if they had already thought about it or had seen it somewhere before the experiment (i.e., “I cut my birthday cake with a key last year because I didn’t have a knife” or “I saw the main character cut the cake with a key in my favorite movie”).

#### Scoring.

Episodic Responses during ESI and control interviews were coded following the same protocol as in Experiment 1. The same two raters from Experiment 1 coded 3 participants showing medium-high IC for episodic specific ideas (IC = .9) and general ideas (IC = .71), and the first rater continued rating the rest of the sample.

The dimensions of Alternative Uses Test were scored following the same protocol as Experiment 1, except from a slightly modification of the definition of appropriateness as feasible and useful, based on Addis et al. [79]. The first rater from Experiment 1 evaluated every AUT dimension after showing higher IC with a new rater who coded six participants chosen randomly from the sample: flexibility (IC = .98), originality (IC = .81), appropriate uses (IC = .95), categories of appropriate uses (IC = .89) and elaboration (IC = .94).

### Results

#### Interview.

The raw number of details generated during the ESI and control interviews are summarized in Table 7. Since there were almost no external episodic details during the ESI (M = 0; SD = 0) or control interviews (M = 0.71, SD = 0.75), these data were excluded from the analysis.

**Table 7:** Average (SD in brackets) of episodic specific and general details by type of interview.

Type of Interview	Episodic Specific Details	General Details
**ESI**	17.38 (2.95)	2.33 (2.88)
**Control**	4.13 (2.59)	14.13 (3.19)

We conducted an ANOVA to assess the effect of interview type (ESI vs. control) on the proportion of episodic specific details over the total amount of details per condition (for consistency with Experiment 1 analysis). The results of this analysis revealed a main effect of interview type, *F*(1, 23)= 706; *p* < .001; η^2^_p_ = 0.968, indicating that children produced more episodic specific details in the ESI condition (M = 17.38: SD = 2.95) than in the control condition (M = 4.13; SD = 2.59) (see Table 8).

**Table 8:** Average (SD in brackets) of the proportion of episodic specific and general details over total amount of details per condition.

Type of Interview	Proportion of Episodic Specific Details	Proportion of General Details
**ESI**	0.89 (0.1)	0.11 (0.1)
**Control**	0.2 (0.1)	0.76 (0.11)

#### Divergent thinking: Alternative uses test.

Results on the ANOVAS performed on each of the six divergent thinking dimensions showed no significant differences between the ESI and control interviews: fluency, *F*(1,23)=1.03, *p* = .322, η^2^_p_=0.012, flexibility, *F*(1,23)=0.479, *p* = .479, η^2^_p_=0.020, appropriate uses, *F*(1,23)=0.385, *p* = .536, η^2^_p_=0.017, categories of appropriate uses, *F*(1,23)=1.18, *p* = .289, η^2^_p_=0.049, originality, *F*(1,23)=2.93, *p* = .101, η^2^_p_=0.113, and elaboration, *F*(1,23)=0.042, *p* = .840, η^2^_p_=0.002 (See Table 8).

Since the results of the ANOVAs did not yield a significant effect in any dimension, we decided to perform Bayesian analyses (see Table 9) to assess the extent to which our data supported the null hypotheses using the default parameters in JASP 0.16.2.0. [80]. The BF01 value represents the strength of the evidence that data provide for the null model (H0). Results revealed moderate evidence in favor of the null hypothesis in the dimensions of elaboration and anecdotal evidence for the remaining dimensions (see [81]) (the most relevant results from Experiment 1 and 2 are summarized in S7 Appendix in S1 File).

**Table 9:** Means and standard deviations (in brackets) by type of induction in alternative uses test (AUT) dimensions and Bayes Factor.

	ESI	Control	BF01
Fluency	22.2 (14.1)	23.5 (15.7)	2.31
Flexibility	3.97 (1.83)	4.11 (1.85)	2.80
Appropriate Uses	15.7 (9.33)	16.5 (9.93)	2.97
Categories of Appropriate Uses	3.06 (1.44)	3.30 (1.30)	2.25
Originality	2.71 (0.52)	2.62 (0.56)	1.13
Elaboration	1.66 (0.48)	1.65 (0.49)	3.48

Finally, when the counterbalancing order of the interviews was included as a between subjects variable in the ANOVA, it did not alter the results. The main effect of order and its interactions with type of interview was non-significant for all AUT dimensions (all ps> .05).

#### Old vs. new responses.

In addition, we conducted a repeated measures ANOVA to examine whether the effect of induction type (ESI vs. control) modulated the type of generated ideas (old vs. new ideas). Results revealed a marginal main effect of type of idea, *F*(1,23)=3.805, *p* = .063, η^2^_p_=0.142 indicating that more uses were rated as new (ESI: M = 13.6; SD = 11.9; Control: M = 14.5, SD = 14.2) than as old (ESI: M = 8.58, SD = 5.27; Control: M = 9, SD = 7.07). The main effect of type of interview *F*(1,23)=1.025, *p* = .322, η^2^_p_ = 0.043, and the interaction between type of interview x type of idea were not significant *F*(1,23)=0.068, *p* = .797, η^2^_p_=0.003.

### Discussion

The overall aim of Experiment 2 was to investigate episodic specific recall during ESI in fifth-grade children using non-adapted videos previously employed by Madore et al. [31]. These materials were expected to be less engaging and more difficult to recall. During the memory interview, children recalled more episodic details following ESI compared to the control condition, indicating that ESI effectively enhanced episodic specificity. However, ESI had no effect on any dimensions of the divergent thinking task (AUT) compared to the control condition, as confirmed by Bayesian analyses. Thus, although the non-adapted material presented during ESI improved episodic memory during the interview compared to the control condition, this effect did not transfer to divergent thinking performance (see [38] for similar findings).

In addition, the number of old ideas (retrieved from memory) and new ideas (generated for the first time during the experiment) was measured during the AUT. Children reported a marginally higher number of new ideas than old ideas in both the ESI and control conditions. This contrasts with findings from Madore et al. [37], where adults reported more ‘old’ than ‘new’ ideas. These results may reflect that developmental differences in the type of ideas generated, possibly due to a greater reliance on episodic memory in adults. However, they may also stem from children’s less efficient reality monitoring (i.e., the ability to distinguish between remembered and imagined events) [82,83]).

## General discussion

This study examines developmental differences between children and adults in the effects of the standard Episodic Specificity Induction (ESI) on episodic memory and creativity using either children-adapted (Experiment 1) or non-adapted (Experiment 2) materials.

Previous research, such as Tanguay et al. [38], found that children generated internal (“on task”) details during an adapted, future-oriented ESI interview, and following the ESI manipulation, suggesting potential benefits for episodic recall, particularly with children-adapted materials. However, no transfer effects to other cognitive tasks requiring episodic memory were observed, possibly due to the use of a future-oriented ESI interview rather than the standard ESI procedure, which focuses on retrieving past information. In this context, the present study investigated whether the beneficial effects of ESI on divergent thinking, previously observed in adults by Madore et al. [36,37], could also be observed in children. To this end, the standard ESI procedure was adapted to better support children’s episodic memory, with the expectation to first increase their episodic recall during the ESI interview and, subsequently, the number of ideas produced in the AUT task.

### Episodic specific recall in children

In Experiment 1, we first observed that overall, children (second and fifth graders) produced less episodic specific details compared to young adults. This finding is consistent with previous studies showing that 9- and 11-year-old children and adults provided higher levels of episodicity in their narratives compared to 5- and 7-year-old children [25,26]. However, this episodic advantage for adults was only apparent when considering the raw number of details. Consistent with Coughlin et al. [26], the total verbal production of the adults was much greater than that of the young children in both ESI and control interviews. However, when we considered the proportion of episodic details in participants’ total production per condition, the difference between children and adults during ESI disappeared, suggesting that retrieval was facilitated by the use of child-relevant material. Interestingly, the results also indicated that participants of all ages, regardless of the materials (Experiments 1 and 2), were influenced by the ESI interview and produced more episodic specific retrieval during the ESI interview than in the control condition. Thus, the ESI procedure was successful in orienting recall towards the episodic details in the video, and this was true for all age groups. This finding is consistent with the results by Tanguay et al. [38], as they found that 6- and 7-year-old children also showed an increased number of internal (“on task”) details recalled during an episodic future-oriented ESI compared to a control condition.

In addition, the results of Experiment 1 with materials adapted for children showed that although adults produced more episodic specific details than children, developmental changes disappeared when the total number of productions during ESI was considered. Therefore, it is possible that if ESI involves retrieval of past information for which children have more familiarity (TV cartoons), their episodic memory may be enhanced, thereby reducing differences between children and adults in both the number of episodic details. These results are consistent with previous developmental studies that demonstrated that when children can rely on their prior knowledge (i.e., schema knowledge) to provide conceptual regularities to integrate other events, their memory increases [48]. These results point to the importance of providing child-appropriate materials to improve memory for past events.

### Effect of ESI on divergent thinking: AUT

Nevertheless, in contrast to previous findings in which ESI improved performance on subsequent cognitive tasks such as divergent thinking tasks (i.e., AUT; [36,37]), the present study does not clearly support this assumption. In Experiment 1, participants produced fewer ideas and categories of ideas following ESI compared to the control condition, although their ideas were more elaborated after ESI. However, in Experiment 2, no significant differences emerged between conditions across any of the AUT dimensions.

Several factors may explain the lack of replication of previous ESI effects on the AUT observed in earlier studies with adults [36,37]. First, the enhanced episodic recall observed in Experiment 1, where participants watched children-adapted videos (i.e., engaging cartoons and an easier narrative story), may have contributed to greater fluency in the control condition during AUT task, which required participants to reflect on an entertaining video without a cognitively demanding retrieval process, may have induced a more positive mood, thereby increasing idea fluency and flexibility. This aligns with previous research showing that positive mood enhances performance in divergent thinking tasks [74–78]. In addition, the increase in fluency and flexibility in the control condition, presumably resulted in less elaboration in the control condition (when compared to the ESI condition). Thus, it is possible that participants following ESI elaborated their ideas more at the cost of a significantly lower number of ideas (see [71], for a similar pattern). In contrast, children in Experiment 2, who watched non-adapted videos, showed no benefit from ESI in either fluency, flexibility, or elaboration. Bayesian analyses further supported the absence of differences between conditions. These findings are consistent with Tanguay et al. [38], who also failed to observe transfer from an adapted ESI to other cognitive tasks. Thus, although the non-adapted materials presented during ESI in Experiment 2 improved episodic memory during the interview, this improvement was not strong enough to transfer to the divergent thinking task. Moreover, the neutral nature of the standard videos from Madore et al. [31] support that no mood-induction occurred following the control interview.

Another factor that should be considered when interpreting these results is the role of cultural context in how ESI influences divergent thinking. In this regard, both experiments in the present study were conducted with Spanish participants, in comparison to the studies by Madore et al. [36,37,84] in the U.S. In line with this, Ahmed et al. [68] conducted a study in Leeds (United Kingdom) examining the effects of ESI and the same control interview on young and older adults using standard ESI materials. Their findings showed no significant effect of ESI on any AUT dimensions. Similarly, Purkart et al. [85] found no evidence that ESI influenced the AUT number of categories of appropriate uses in Canadian older adults. These findings suggest that cultural differences may modulate the effects of ESI, potentially explaining differences between our study and Madore et al. [36,37] findings with American participants.

Additionally, future research should examine the effects of standard ESI on children by incorporating other tasks that explicitly relies on episodic memory, such as the imagination task used by Madore et al. [36,37]. This would help determine whether ESI facilitates performance on this imagination task but not on the AUT. In addition, as recently stated by Ahmed et al. [68], including a no-induction baseline condition would clarify whether the observed effects are due to ESI itself, or to the nature or relative cognitive demands of the control interview using children-adapted material. This comparison would help disentangle the unique contribution of ESI. Finally, future studies could include participants across a broader age range, from childhood to adolescence, to assess how the effects of ESI evolve with development, and might also consider including gender as an independent variable to explore potential interaction effects.

## Conclusions

In conclusion, this study provides evidence for developmental differences between children and adults in episodic specificity during ESI, a procedure designed to improve performance on a variety of cognitive tasks involving episodic thinking [33]. In addition, we showed that different audiovisual materials used during episodic induction can differentially influence participants’ episodic recall and their performance on subsequent creativity tasks. This study opens new research interest in which factors moderate the effect of ESI on children’s ability to generate novel and useful ideas in creativity tasks, which may have significant implications for educational settings.

## Supporting information

S1 File Supporting Information. This file contains: S1 Appendix. Inductions’ protocol in Experiment 1 and 2. S2 Appendix. Tables with RAT items for children and young adults. S3 Appendix. ANOVA and descriptive data for the effect of induction according to setting differences (in-person vs. online) in Experiment 1. S4 Appendix. AUT instructions. S5 Appendix. Scoring protocol for interviews. S6 Appendix. Table with means, standard deviations and p-values in the AUT dimensions including and not including common uses in the analysis. S7 Appendix. Summary tables of the main results for episodic recall and divergent thinking (AUT) in Experiments 1 and 2.
(DOCX)
