# Correction: Exploring episodic specificity induction on divergent thinking in children

**DOI:** 10.1371/journal.pone.0348812

**Published:** 2026-04-30

**Authors:** The PLOS One Staff

There are errors in the Funding statement. The correct Funding statement is as follows: This work was supported by grant PID2024-15977NB-I00 and PID2021-127728NB-I00 by the Ministry of Science, Innovation and Universities MICIU/ AEI/10.13039/501100011033 (https://www.aei.gob.es/sobre-aei), by the European Regional Development Fund ERDF/EU (https://ec.europa.eu/regional_policy/funding/erdf_en) to TB and AM, the grant FPU20/01883 by Spanish Ministry of Universities to GT (https://www.ciencia.gob.es/Convocatorias/2024/FPU2024.html), and PGC2018-093786-B-I00, Projects I+D+i Junta de Andalucia- Feder Funds P20-00107 and BCS.384-UGR20 from Junta de Andalucia (https://www.juntadeandalucia.es/organismos/fomentoarticulaciondelterritorioyvivienda/servicios/proyectos-idi.html) to TB. The Mind, Brain and Behavior Research Center receives funding from grants CEX2023-001312-M by MCIN/AEI/10.13039/501100011033 (https://investigacion.ugr.es/recursos-humanos/otras-convocatorias/predoctorales-mdm-eng) and UCE-PP2023-11 (https://investigacion.ugr.es/ayudas/plan-andaluz/feder-2023) by the University of Granada. The funders did not play any role in the study design, data collection and analysis, decision to publish or preparation of the manuscript.

The publisher apologizes for the error.

## References

[1] Tomás G, Bajo T, Marful A. Exploring episodic specificity induction on divergent thinking in children. PLoS One. 2026;21(3):e0341294. doi: 10.1371/journal.pone.0341294 41790759 PMC12965567

