# Supplementary material for: Exploring episodic specificity induction on divergent thinking in children
Source: PLoS One. 2026 Mar 6;21(3):e0341294. doi: 10.1371/journal.pone.0341294 (PMC12965567; doi:10.1371/journal.pone.0341294)
Supplement: S1 File — This file contains: S1 Appendix. Inductions’ protocol in Experiment 1 and 2. S2 Appendix. Tables with RAT items for children and young adults. S3 Appendix. ANOVA and descriptive data for the effect of induction according to setting differences (in-person vs. online) in Experiment 1. S4 Appendix. AUT instructions. S5 Appendix. Scoring protocol for interviews. S6 Appendix. Table with means, standard deviations and p-values in the AUT dimensions including and not including common uses in the analysis. S7 Appendix. Summary tables of the main results for episodic recall and divergent thinking (AUT) in Experiments 1 and 2. (DOCX) [file pone.0341294.s001.docx]

**S1 File. Supporting Information**

**S1 Appendix: Inductions’ protocol in Experiment 1 and 2**

- **Experiment 1**

Two short clips from “Bandolero” (TV cartoons). Available upon request.

1. **Episodic Specificity Induction Interview Protocol**

We provide the original Spanish version followed by the English translation

- 1. **Introduction (English translation/Spanish)**

So now I’m going to ask you a few questions about the video you watched. I haven’t seen the video myself, so you’re the expert on that. I will also use an audio-recorder and write down what you say to keep track if that’s okay. How does that sound to you?/ Ahora te voy a hacer una serie de preguntas sobre el video que has visto. Yo no he visto el vídeo así que tú eres el/la experto/a. También grabaré tus respuestas si te parece bien.

- 1. **Mental imagery about the surroundings**

Okay, so first I want you to close your eyes and get a picture in your head about the surroundings of the video you watched. I want you to think about what types of things were in the environment and how they were arranged and what they looked like. Once you have a really good picture in your head I want you to tell me everything you remember about the surroundings. Try to be as specific and detailed as you can. / Cierra los ojos e imagina el paisaje del video que acabas de ver. Quiero que pienses en las cosas que había en el entorno, si cambiaba de sitio, etc. Una vez que tengas en tu cabeza una buena imagen del paisaje quiero que me cuentes todo lo que puedas recordar. Intenta recordar tantos detalles como puedas.

- Tell me more about… (details mentioned) / Cuéntame más de… (detalles mencionados)
- Tell me more about how the places in the video./ Cuéntame más sobre los distintos lugares que había.
- Tell me more about the furniture in the video. / Cuéntame más sobre los muebles que había en la cueva.
- Tell me about the animals in the video / Háblame de los animales que se ven en el vídeo.
- Tell me more about the houses (if mentioned) / Háblame de las casas que aparecen (si las menciona).
- How was the sky? / ¿Cómo era el cielo?
  1. **Mental imagery about the people**

Now I want you to close your eyes and get another picture in your head, this time about the people in the video you watched. I want you to think about what the people looked like and what they were wearing. Once you have a really good picture in your head I want you to tell me everything you remember about the people in the video. Again, try to be as specific and detailed as you can. / Cierra los ojos e imagina los personajes que aparecían en el video que acabas de ver. Quiero que pienses en cómo eran, cómo hablaban y la ropa que llevaban. Una vez que tengas en tu cabeza una buena imagen de cada uno de ellos quiero que me cuentes todo lo que puedas recordar. Intenta recordar tantos detalles como puedas.

- Tell me more about (details mentioned) / Cuéntame más sobre (detalles mencionados).
- Tell me more about the (character)’s outfit. / Cuéntame más detalles sobre la ropa de (personaje determinado)
- How was the hair of (mentioned character)? / ¿Cómo era el pelo de (personaje determinado)?
  1. **Mental imagery of actions**

Now I want you to close your eyes and get a picture in your head about the actions in the video you watched. I want you to think about what the people were actually doing in the video and how they did these things. Once you have a really good picture in your head I want you to tell me everything you remember about the actions starting with the first one and ending with the last one. Try to be as specific and detailed as you can. / Ahora quiero que cierres los ojos y que pienses en las acciones que realizan los personajes del video. Quiero que imagines lo que cada personaje estaba haciendo realmente y cómo lo hacían. Una vez lo tengas en tu cabeza una buena imagen de esto quiero que me cuentes todo lo que puedas recordar. Intenta recordar tantos detalles como puedas.

- Tell me more about… (actioned mentioned) / Háblame más de (acción mencionada).

(Only if particpant doesn’t give sequence of actions first time around/Si el sujeto no menciona las cosas de forma secuenciada).

- What happened after that? What was the next thing? / ¿Qué pasaba antes de (acción)? ¿Y después?
- What was the last thing that happened? / ¿Qué fue lo último que pasó en el vídeo?

1. **Control Interview Protocol**
   1. **Introduction**

So now I’m going to ask you a few questions about the video you watched. I’m also going to use the audio-recorder and write down what you say to keep track if that’s okay. How does that sound to you? / Ahora te haré algunas preguntas sobre el video que has visto. También usaré la grabadora si te parece bien.

- 1. **Question bank**
- First I want you to tell me what you thought about the video / Lo primero que voy a preguntarte es, ¿qué te ha parecido el vídeo?
- Did you like it? / ¿Te ha gustado?
- What did you like the most? Did you dislike something? / ¿Qué es lo que más te ha gustado del vídeo? ¿Qué es lo que menos te ha gustado del vídeo?
- Do you thing the video was too long? Would you like to see more? / ¿Te ha parecido muy largo el vídeo? ¿Te habría gustado ver más?
- What do you think about the characters in the video? / ¿Qué te han parecido los lugares que salían en el vídeo?
- Which character was your favorite? Which character did you like the least? / ¿Qué te han parecido los lugares que salían en el vídeo?
- Which title would you give for this video? / ¿Puedes decirme qué título pondrías para este video?
- Do you think this video was old? When do you think this video was made? Would you like to see this series on TV? / ¿Te ha parecido antiguo? ¿Cuándo piensas que se hizo este vídeo? ¿Te gustaría que lo echasen por la tele?
- Do you think this cartoons were hand-drawn or made by computer? / ¿Crees que los dibujos de esta historia se hicieron a mano o por ordenador?
- Can you guess the people’s occupations based on the video? / ¿En qué crees que trabaja cada personaje?
- Have you ever seen a series like this? / ¿Ves alguna serie que se parezca a esta?
- Could you draw some cartoons like these? / ¿Podrías hacer dibujos parecidos a los de esta serie?
- Do you think your parents would like this show? And your friends? And family? / ¿Piensas que les gustaría a tus padres esta serie? ¿Y a tus amigos?
- Anything else you would like to comment about this video? / ¿Algo más que quieras comentar sobre el video?
- **Experiment 2**

Videos from Madore et al. (2014)

1. **Episodic Specificity Induction Interview Protocol**

Almost identical to the one in S1 Appendix, with slight differences like questions about the surroundings (e.g. tell me more about the objects in the kitchen, were there any other room?) or people (e.g. how was the face of these people?) according to these videos.

1. **Control Interview Protocol**
   1. **Introduction**

So now I’m going to ask you a few questions about the video you watched. I’m also going to use the audio-recorder and write down what you say to keep track if that’s okay. How does that sound to you? First I want you to tell me what you thought about the video. I don’t want you to give me a summary of it. Just tell me what your thoughts and opinions of it were. What were your general impressions of the video? / Ahora te voy a hacer una serie de preguntas sobre el vídeo que acabas de ver. Yo no he visto el video así que tú eres el/la experto/a de este video. También voy a usar la grabadora y escribir algunas notas sobre lo que digas si te parece bien. ¿Qué te parece? Primero me gustaría que me cuentes qué has pensado sobre este video. No quiero que me hagas un resumen de este, simplemente cuéntame sus pensamientos y opiniones sobre este. ¿Cuáles han sido tus impresiones generales sobre este video?

- 1. **Question Bank**
- What adjectives would you use to describe the setting of the video? The people? The actions? / ¿Qué adjetivos usarías para describir el entorno de este video? ¿Y para la gente? ¿Y para las acciones de lo que hacían?
- Did you have any other opinions about the setting of the video? Did you have any other opinions about the people? The actions? / ¿Tienes alguna otra opinión sobre el lugar donde se rodó este video? ¿Y sobre la gente? ¿Y sobre las acciones?
- Did you like the video? Why? / ¿Te ha gustado el video? ¿Por qué?
- When do you think this video was made? / ¿Cuándo crees que se rodó este vídeo? ¿Por qué?
- How do you think it was made (what equipment do you think they used?) / ¿Cómo crees que se rodó este video? ¿Qué equipo crees que fue utilizado para grabarlo?
- Did the video remind you of anything from your own life? / ¿Te recordó este vídeo a algo de tu propia vida?
- Can you guess how big the place was based on the video? / ¿Podrías adivinar cómo de grande es el espacio donde sucede el vídeo?
- Can you guess the people’s occupations based on the video? / Basándote en el vídeo, ¿sabrías decirme cuales crees que son las ocupaciones o el trabajo de las personas que aparecen?
- Were there any other thoughts or opinions you had about the video? Is there anything else you wanted to say about it? / ¿Alguna otra idea u opinión que tengas sobre el vídeo? ¿Algo más que quieras decir?

**S2 Appendix. Tables with RAT items for children and young adults**

| **Table S1***.* **RAT for second and fifth graders** | |
| --- | --- |
| **Items set A (answer)** | **Items set B (answer)** |
| Colchón, edredón, habitación (cama) | Bola, esquí, muñeco (nieve) |
| Leyenda, infancia, hada (cuento) | Borrego, vaca, sabrosa (carne) |
| Oscuridad, estrellada, luna (noche) | Comida, redonda, tabla (mesa) |
| Pata, cómodo, sentarse (silla) | Uva, borracho, bodega (vino) |
| Oxígeno, sombra, tronco (árbol) | Globos, bailar, cumpleaños (fiesta) |
| Encía, cepillo, dentista (diente) | Volar, rapidez, piloto (avión) |
| Bolígrafo, sobre, recibir (carta) | Naturaleza, tranquilidad, hierba (campo) |
| Agujas, tictac, despertador (reloj) | Arcoíris, pintura, amarillo (color) |
| Flor, espina, bella (rosa) | Respirar, fresco, viento (aire) |
| Blanco, arroz, anillos (boda) | Teatro, estrella, película (actor) |
| Dedal, gordo, meñique (dedo) | Diversión, ritmo, moverse (baile) |
| Bosques, espaldas, meta (guarda) | Fuego, plumas, uñas (corta) |
| Caídas, choques, rayos (para) | Frutas, platos, vajillas (lava) |
| Manchas, miedos, esmalte (quita) | Moscas, ratas, suegras (mata) |
| Corchos, muelas, puntas (saca) | Manteles, pantallas, vidas (salva) |
| Gotas, kilómetros, revoluciones (cuenta) | Cabezas, hielos, olas (rompe) |
| Hombre, oferta, mercado (super) | Chimeneas, cristales, parabrisas (limpia) |
| Masculino, femenino, opuesto (sexo) | Descafeinado, solo, cortado (café) |
| Reproductor, circulatorio, eléctrico (aparato) | Mediterránea, saludable, equilibrada (dieta) |
| Sangre, gemelo, mayor (hermano) | Cascabel, pitón, marina (serpiente) |

| **Table S2. RAT for young adults** | |
| --- | --- |
| **Items set A (answer)** | **Items set B (answer)** |
| Centímetro, medir, compresa (regla) | Caricia, escribir, guantazo (mano) |
| Comida, redonda, table (mesa) | Negro, bolsillo, ozono (agujero) |
| Borrego, vaca, sabrosa (carne) | Puente, flecha, romano (arco) |
| Planeta, plantas, arena (tierra) | Arcoíris, pintura, amarillo (color) |
| Metal, candado, abrir (llave) | Camino, acerca, ciudad (calle) |
| Hoja, ojos, césped (verde) | Pequeño, casas, campesinos (pueblo) |
| Gris, Tabaco, tos (humo) | Sillón, televisión, alfombra (salón) |
| Consulta, curar, hospital (doctor) | Blanco, arroz, anillos (boda) |
| Romántica, cantar, melodía (canción) | Dedal, gordo, meñique (dedo) |
| Molestar, desagradable, sonido (ruido) | Naturaleza, tranquilidad, hierba (campo) |
| Playa, palmera, barco (isla) | Bonito, chalet, flores (jardín) |
| Estructura, botellón, espectáculo (macro) | Portada, punto, peso (contra) |
| Maletas, lámparas, aviones (porta) | Consola, cámara, club (video) |
| Móvil, servicio, lavado (auto) | Valorado, rojo, mundo (infra) |
| Pensado, aventurado, venido (bien) | Arriba, manga, bajo (boca) |
| Razón, sabor, sentido (sin) | Tensión, calórico, ventilado (hiper) |
| Hombre, oferta, mercado (super) | Carro, sierra, bomba (moto) |
| Justo, especial, familiar (precio) | Noble, maciza, tallada (madera) |
| Partido, representante, discurso (político) | Luz, desastre, entorno (natural) |
| Reproductor, circulatiorio, eléctrico (aparato) | Diplomática, contrarreloj, universitaria (Carrera) |

**S3 Appendix. ANOVA and descriptive data for the effect of induction according to setting differences (in-person vs. online) in Experiment 1**

A 2 (condition: ESI vs. control) x 2 (setting: in-person vs. online) ANOVA revealed that there were not differences between the online and in-person version of the experiment in any of the dependent variables (no significant main effect of setting nor interaction setting x condition, all ps > .05). Means (SD) for the seven in-person participants across dependent variables: raw number of episodic specific details: ESI=31.4 (4.50); control=3 (3.06); fluency: ESI=19.1 (5.08), control =21.1 (9.03); flexibility: ESI=4.67 (1.52), control=4.43 (0.85); appropriate uses: ESI=18.1 (5.27), control=20.3 (9.20); categories of appropriate uses: ESI= 4.10 (0.94), control=4.52 (1.5); originality: ESI = 2.60 (0.46), control = 2.83 (0.32); elaboration: ESI=1.39 (1.38), control=1.34 (1.31).

**S4 Appendix. AUT instructions**

1. **AUT instruction in Experiment 1**

“Now, I will show you everyday objects, and you will have to come up with as many possible uses as possible for each one. For example, a “brick” is typically used for construction, but you could use a brick as a pillow, as a nutcracker or to play with many of them and to make a “domino effect”. You will have 2 minutes for each object. You can mention its common use, but try to focus on unusual uses, ones that are different from the typical function and distinct from one another ¿Any question?”

1. **AUT instruction in Experiment 2**

“Now, I will show you everyday objects, and you will have to come up with as many possible uses as possible for each one. For example, a “brick” is typically used for construction, but you could use a brick as a pillow, as a nutcracker or to play with many of them and to make a “domino effect”. You will have 2 minutes for each object. Try to generate as many uses trying to focus on unusual uses, ones that are different from the typical function ¿Any question?”

**S5 Appendix. Scoring protocol for interviews**

Independent judges received the scoring protocol below:

1. **Segmentation**

The first part of the procedure consists of segmenting the interview into pieces of information or details. We define a detail as a "single occurrence, observation, or thought, typically expressed as a grammatical clause." Any information added to that clause will be considered as a new detail. Some of the rules for this segmentation are:

- Consider separately the pieces of information about places, people, actions, opinions, impressions, autobiographical memories, or guesses in different details.
- Depending on the context, some actions can be paired within the same detail when carried out in a sequence (e.g., "turned on the water to fill the kettle" or "comes and places the saw on the fridge").
- When the participant adds more details related to the same idea, it will count as a new detail (e.g., "the girl has blonde hair," "but her hair was not very long," "she also had a bandana on her head").
- When an opinion is associated with a specific detail of the video, it is separated into 2 different details (e.g., "I think it’s very rude the moment the girl tells the guy he didn’t feed the cat" would be separated into two details, an opinion "I think the girl is very rude" and an action "when she tells the guy he didn’t feed the cat").
- Repeated details are eliminated (directly not transcribed).
- Monosyllabic responses of yes/no or other sounds or gestures are also not transcribed.

1. **Categorization**

To categorize the details from the interviews, we now have the list of transcribed details mixed up so as not to know the condition. If there are monosyllabic details like "no" or "yes", you can eliminate them and not categorize them. Ideas will be categorized into three categories:

**Specific Episodic (S):** Concrete pieces of information related to places, people, and actions remembered from the video they watched. Example: "there were green trees," "the woman put the flowers in the vase." Do not distinguish between real or false episodic memory of the video, meaning if the idea says, "the woman had a blue shirt" and it was actually white, we would still consider it as specific episodic (S). Do not include overly generic or obvious information in this category (e.g., "they were doing things," "it was a house," "there were people..."). These very generic details are considered as General (G) because they are not specific.

**General (G):** In this category, ¡ semantic information/general knowledge (e.g., "kitchens usually have a sink"), opinions/ impressions (e.g., "it’s rude for her to talk like that"), and hypotheses ("I imagine she used the gloves for cleaning") will be included.

**External Episodic (E):** This category contains episodic pieces of information that are not specific to the video they just watched. For example, when they reveal autobiographical information (e.g., "I remember watching this series when I was a kid"), details from the previous session's video (e.g., "I remember the shirt was the same as in the first video"), or imagination about what will happen next (e.g., "after the video ends, I imagine the mother will get her child back").

**S6 Appendix. Table with means, standard deviations and p-values in the AUT dimensions including and not including common uses in the analysis**

| **Table S3. Means (M) and Standard Deviations (SD) in Alternative Uses Test (AUT) dimensions including and not including common uses in the analysis and p values for the main effect of induction type on AUT** | | | | | | |
| --- | --- | --- | --- | --- | --- | --- |
|  | **With Common Uses** | | | **Without Common uses** | | |
|  | **ESI** | **Control** | ***p* values** | **ESI** | **Control** | ***p* values** |
| Fluency | 22.6 (9.50) | 24.7 (10.5) | *p*=.007 | 20.3 (9.36) | 22.5 (10.3) | *p*=.006 |
| Flexibility | 4.39 (1.41) | 4.78 (1.60) | *p*=.017 | 3.89 (1.42) | 4.31 (1.59) | *p*=.016 |
| AU | 20.4 (8.07) | 22.5 (9.12) | *p*=.002 | 18.2 (8.07) | 20.4 (9.09) | *p*= .002 |
| CAU | 4.17 (1.34) | 4.51 (1.47) | *p*=.026 | 3.67 (1.37) | 4.03 (1.48) | *p*=.021 |
| Originality | 2.85 (0.57) | 2.93 (0.52) | *p*=.195 | 3.06 (0.47) | 3.12 (0.40) | *p*=.244 |
| Elaboration | 1.49 (0.38) | 1.43 (0.35) | *p*=.04 | 1.53 (0.39) | 1.46 (0.39) | *p*=.024 |
| Note: *AU: Appropriate Uses, CAU: Categories of Appropriate Uses | | | | | | |

**S7 Appendix. Summary tables of the main results for episodic recall and divergent thinking (AUT) in Experiments 1 and 2**

| **Table S4. Summary of the 3(age group: second grade, fifth grade, adults) x 2 (type of interview: ESI, control) ANOVA on interview responses** | | | | |
| --- | --- | --- | --- | --- |
| Experiment | Dependent variable | **Main effect of ESI** | **Main effect of Age** | **Interaction Interview x Age** |
| Experiment 1 | Raw number of episodic specific details | *F*(1,69) =1017.5; *p*<.001; η^2^_p_ = 0.936  (Control < ESI) | *F*(2,69) =11.9, *p*<.001; η^2^_p_ = 0.257  (2nd < 5th < YA) | *F*(2,69) =22.4; *p*<.001; η^2^_p_ = 0.393  (Age differences during ESI not control) |
|  | Proportion of episodic specific details | *F*(1,69) = 3013.95; *p*<.001; η^2^_p_ = 0.978  (Control < ESI) | *F*(2,69) =10.9; *p*<.001; η^2^_p_ = 0.239  (YA < 2nd = 5th ) | *F*(2,69) =9.77; *p*<.001; η^2^_p_ = 0.221  (Age differences during control not ESI) |
| Experiment 2 | Proportion of episodic specific details | *F*(1, 23)= 706 ; *p*<.001; η^2^_p_ = 0.968  (Control < ESI) | _ | _ |
| Note: Second graders: 2nd, Fifth graders: 5th, Young adults: YA | | | | |

| **Table S5. Summary of the 3(age group: second grade, fifth grade, adults) x 2 (type of interview: ESI, control) ANOVA on AUT.** | | | | |
| --- | --- | --- | --- | --- |
| Experiment | Dependent variable | **Main effect of Interview** | **Main effect of Age** | **Interaction Interview x Age** |
| Experiment 1 | Fluency | *F*(1,69)=7.62, *p*=.007, η^2^_p_ = 0.099  (ESI < Control) | *F*(2,69)=9.25, *p*<.001, η^2^_p_ = 0.211  (2nd < 5th = YA) | *F*(2,69)=0.329, *p*=.721, η^2^_p_ = 0.009 |
|  | Flexibility | *F*(1,69)=5.51, *p*=.017, η^2^_p_ = 0.08  (ESI < Control) | *F*(2,69)=10.6, *p*<.001, η^2^_p_ = 0.235  (2nd < 5th = YA) | *F*(2,69)=0.85, *p*=.432, η^2^_p_ = 0.024 |
|  | Appropriate Uses | *F*(1,69)=10.255, *p*=.002, η^2^_p_ = 0.129  (ESI < Control) | *F*(2,69)=18.1, *p*<.001, η^2^_p_ = 0.344  (2nd < 5th = YA) | *F*(2,69)=0.251, *p*=.778, η^2^_p_ = 0.007 |
|  | Categories of Appropriate Uses | *F*(1,69)=5.191, *p*=.026, η^2^_p_ = 0.07  (ESI < Control) | *F*(2,69)=13.7, *p*<.001, η^2^_p_ = 0.284  (2nd < 5th = YA) | *F*(2,69)=0.209, *p*=.812, η^2^_p_ = 0.006 |
|  | Originality | *F*(1,69)=1.71, *p*=.195, η^2^_p_ = 0.024 | *F*(2,69)=3.74, *p*=.029, η^2^_p_ = 0.098  (2nd < YA) | *F*(2,69)=0.138, *p*=.872, η^2^_p_ = 0.004 |
|  | Elaboration | *F*(1,69)=4.39, *p*=.04, η^2^_p_ = 0.06  (Control < ESI) | *F*(2,69)=5.48, *p*=.006, η^2^_p_ = 0.137  (2nd = 5th <YA) | *F*(2,69)=1.62, *p*=.205, η^2^_p_ = 0.045. |
| Experiment 2 | Fluency | *F*(1,23)=1.03, *p*=.322, η^2^_p_=0.012 | _ | _ |
|  | Flexibility | *F*(1,23)=0.479, *p*=.479, η^2^_p_=0.020 | _ | _ |
|  | Appropriate Uses | *F*(1,23)=0.385, *p*=.536, η^2^_p_=0.017 | _ | _ |
|  | Categories of Appropriate Uses | *F*(1,23)=1.18, *p*=.289, η^2^_p_=0.049 | _ | _ |
|  | Originality | *F*(1,23)=2.93, *p*=.101, η^2^_p_=0.113 | _ | _ |
|  | Elaboration | *F*(1,23)=0.0416, *p*=.840, η^2^_p_=0.002 | _ | _ |
| Note: Second graders: 2nd, Fifth graders: 5th, Young adults: YA | | | | |
